# Diallel genetic analysis for multiple traits in eggplant and assessment of genetic distances for predicting hybrids performance

**DOI:** 10.1371/journal.pone.0199943

**Published:** 2018-06-27

**Authors:** Prashant Kaushik, Mariola Plazas, Jaime Prohens, Santiago Vilanova, Pietro Gramazio

**Affiliations:** 1 Instituto de Conservación y Mejora de la Agrodiversidad Valenciana, Universitat Politècnica de València, Valencia, Spain; 2 Instituto de Biología Molecular y Celular de Plantas, Consejo Superior de Investigaciones Científicas-Universitat Politècnica de València, Valencia, Spain; Università Politecnica delle Marche, ITALY

## Abstract

Evaluation and prediction of the performance of hybrids is important in eggplant (*Solanum melongena*) breeding. A set of 10 morphologically highly diverse eggplant parents, including nine inbred *S*. *melongena* and one weedy *S*. *insanum* accessions, were intercrossed according to a half-diallel mating design without reciprocals to obtain 45 hybrids. Parents and hybrids were evaluated for 14 morphological and agronomic conventional descriptors and 14 fruit morphometric traits using Tomato Analyzer. Genetic distances among parents were estimated with 7,335 polymorphic SNP markers. Wide ranges of variation and significant differences were observed in the set of 55 genotypes for all traits, although the hybrids group had significantly higher vigour and yield than parents. General and specific combining abilities (GCA and SCA) were significant for most (GCA) or all (SCA) traits, although a wide variation was obtained for GCA/SCA ratios. Many relevant traits associated to vigour and yield had low GCA/SCA ratios and narrow-sense heritability (*h*^*2*^) values, while the reverse occurred for most fruit shape descriptors. Broad-sense heritability (*H*^*2*^) values were generally high, irrespective of GCA/SCA ratios. Significant correlations were found between traits related to size of leaf, flower and fruit, as well as among many fruit morphometric traits. Genetic distances (GD) among parents were coherent with their phylogenetic relationships, but few significant and generally low correlations were found between GD and hybrid means, heterosis or SCA. The results provide relevant information for developing appropriate strategies for parent selection and hybrid development in eggplant and suggest that GD among parents have limited value to predict hybrid performance in this crop.

## Introduction

Eggplant (*Solanum melongena* L.) is an important vegetable crop of tropical and subtropical regions of the world, being cultivated in more than 1.79 million ha and having a global production of 51.28 million tons [1]. Eggplant production has increased by 50% in the last decade and the demand is expected to increase, in part due to its high content in bioactive compounds beneficial for human health [2,3]. Despite its economic importance, eggplant breeding has lagged behind other major vegetable solanaceous crops like tomato or pepper [4,5]. Due to the swift growth of human population and increased demand of vegetables coupled with limited availability of cultivable land, it is necessary to develop improved vegetable cultivars to increase yields and meet the demands of consumers [6]. The use of heterosis for yield and other traits of agronomic interest in F1 hybrids has made, and can continue making, major contributions to developing new vegetable crop varieties with improved yield and other characteristics of agronomic interest [7,8]. In this respect, the productive advantages of hybrids in eggplant are known from long time ago [9,10]. Although hybrid breeding in eggplant is expanding and many new F1 hybrid varieties are available [11], a large part of the production still relies in non-hybrid varieties [12].

Eggplant is mostly autogamous [5,13,14] and local varieties display low levels of observed heterozygosity for molecular markers [15–17]. Therefore, pure lines are easy to develop through selection within local varieties [18]. Eggplant flowers are large, easy to emasculate and pollinate by hand and each fruit can give a large number of seeds, typically between 200 and 2000 seeds [19,20]. In addition, male-sterility systems have been described [21,22], which might facilitate the development of hybrids.

Selection of parents giving good hybrids is a critical step for hybrid breeding programs [23,24]. The identification of parents with good general combining ability (i.e., generally giving good hybrids), as well as specific combinations of parents that result in exceptionally good hybrids, allows breeders selecting parents for obtaining hybrids [25–27]. Among the available biometrical procedures for determining general combining ability (GCA) and specific combining ability (SCA), as well as the nature and magnitude of gene actions and heritability of traits, the diallel analysis proposed by [25] has been widely used in different types of crops [28–32]. In this analysis, GCA is due to additive effects and additive × additive interactions, while SCA to dominance effects and additive × dominant and dominant × dominant interactions [27]. Among the different types of diallel crosses, the half-diallel cross including one-directional crosses makes the overall layout more manageable for breeders than with a doubled number of reciprocal crosses with the full diallel analysis [33].

Several studies have used a variable number of parents (four to 10) for half-diallel analysis to evaluate GCA and SCA for yield and several traits of agronomic interest in different eggplant parents and their hybrids from the Mediterranean region [34], southeast Asia [35,36], or Africa [37]. These works have revealed that both GCA and SCA are generally significant in the parents and hybrids evaluated, although their magnitude and relative importance is variable. However, these works lack general information on several parameters of interest for eggplant breeding, like narrow-sense (*h*^*2*^) and broad-sense (*H*^*2*^) heritabilities, as well as correlations among traits [38], and in addition the number of traits evaluated is limited. Furthermore, up to now there have been no studies in which diallel analyses and molecular marker genotyping are coupled to evaluate the reliability of molecular markers for the selection of eggplant parents giving good hybrids. Although Rodríguez-Burruezo et al. [39] found that genetic distances based on AFLP molecular markers were positively correlated with the yield of hybrids as well as with the heterosis of hybrids, these authors based their conclusions on only 10 hybrids obtained among Spanish local varieties. In other crops, the relationships between genetic distances based on molecular markers and agronomic performance, heterosis and SCA of hybrids has been studied in half-diallel crosses, although results have been contrasting depending on the crop, accessions, markers used, and traits evaluated [28,31,32,40].

In this work we evaluate a large number of traits (28) of interest for eggplant breeding, including conventional descriptors [41–43] and fruit morphometric descriptors using the phenomics tool Tomato Analyzer [44,45] in 10 parents, which is considered as an appropriate number for obtaining valid estimates of genetic parameters [46,47], and their respective 45 hybrids. Parents encompass a wide morphological diversity and different origins, including an edible accession of the weedy ancestor of eggplant (*S*. *insanum* L.) [48]. Experimental hybrids between *S*. *insanum* and *S*. *melongena* have been found to be intermediate between parents in characteristics [43] and with potential for commercial utilization in specific markets. Among the markers available in eggplant, we have used SNPs obtained by a high throughput genotyping-by-sequencing platform, which allows scoring thousands of polymorphic SNPs [17,49], and therefore obtaining reliable estimates of genetic distances among eggplant genotypes. Our approach is unprecedented in eggplant in the combination of a large number of parents, number of traits evaluated, genetic parameters and trait correlations studied, and also in the use of genetic distances for predicting the performance of hybrids, their heterosis and SCA in a half-diallel mating design. The results obtained will provide relevant information for eggplant breeding, in particular for developing new eggplant hybrid varieties.

## Material and methods

### Plant materials

**Fig 1:**
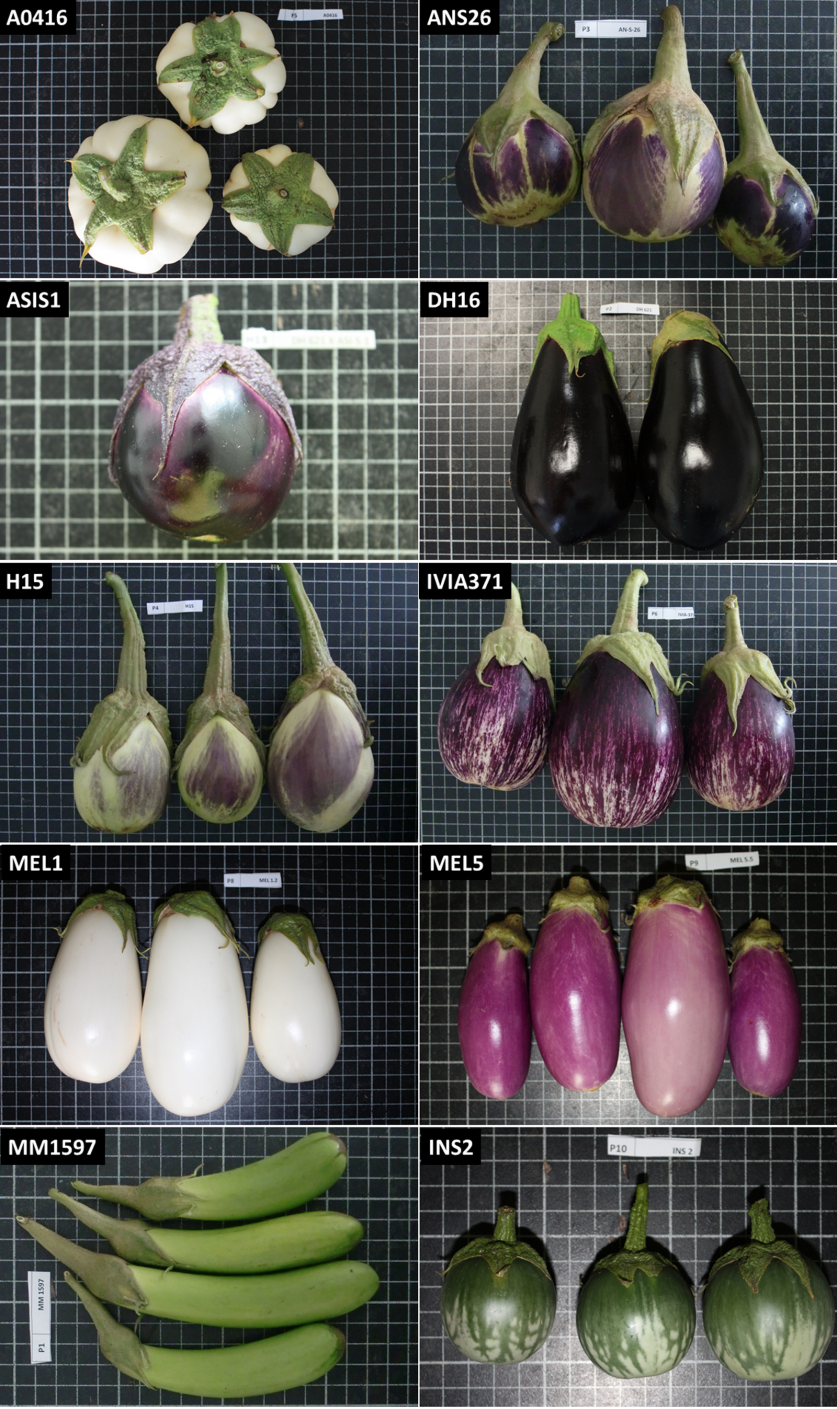
Fruits of the 10 eggplant parentals used in the diallel analysis. Materials include nine cultivated *S*. *melongena* (A0416, ANS26, ASIS1, DH621, H15, IVIA371, MEL1, MEL5, and MM1597) and one weedy *S*. *insanum* (INS2) accessions. Fruits are not depicted at the same scale; the size of the grid cells is 1 cm × 1 cm.

Nine inbred cultivated eggplant (*S*. *melongena*) accessions plus one weedy accession of *S*. *insanum* were used as parents for the present study (Table 1). These accessions were selected based on their differing morphological features, especially in relation to fruit size, shape and colour (Fig 1). The parents used consist of materials from the Occidental and Oriental cultivated eggplant groups [15] including two eggplant accessions (MM1597 and MEL5) from the primary center for diversity in Southeast Asia [50], three (ANS26, H15, and IVIA371) from the Spanish secondary center of diversity [51], one (MEL1) from West Africa, one of unknown origin (A0416), one breeding line (DH621), which is a doubled haploid of the commercial hybrid Ecavi (Rijk Zwaan Ibérica, Almería, Spain), as well as a *S*. *insanum* accession (INS2) originating from Sri Lanka (Table 1). The accession names, their origin and main fruit characteristics are indicated in Table 1. The 10 parental genotypes were intercrossed during the summer season of 2015 using a diallel mating design excluding reciprocals [25] to obtain 45 F1 hybrids.

**Table 1:** Materials of cultivated (*S*. *melongena*) and weedy (*S*. *insanum*) eggplant used in the present study. Information also includes their origin and main characteristics.

Accession	Origin	Group	Fruit size	Fruit shape	Stripes	Primary fruit colour
*S*. *melongena*						
A0416	Unknown	Unknown	Intermediate	Flattened	No	White
ANS26	Spain	Occidental	Intermediate	Obovate	No	Purple
ASIS1	Spain	Oriental	Intermediate	Round	No	Black
DH621	Doubled haploid breeding line	Occidental	Large	Semi long	No	Black
H15	Spain	Occidental	Intermediate	Semi long	No	Purple
IVIA371	Spain	Occidental	Large	Long	Yes	Purple
MEL1	Ivory Coast	Occidental	Intermediate	Semi long	No	White
MEL5	Sri Lanka	Oriental	Intermediate	Semi long	No	Pale purple
MM1597	India	Oriental	Large	Very Long	No	Green
*S*. *insanum*						
INS2	Sri Lanka	Oriental	Small	Round	Yes	Green

### Growing conditions

Seeds of the parents and hybrids were germinated and transplanted into an open field plot situated in the campus of the Universitat Politècnica de València (Valencia, Spain; GPS coordinates of the plot: 39° 28′ 55″ N, 0° 22′ 11″ W; altitude 7 m a.s.l.) on May 2016. A randomized block-design with three replications and three plants was established. Plants were grown using a spacing of 1.2 m between rows and 1.0 m within the row. Irrigation was applied using drip irrigation; fertilization was provided through the irrigation system and consisted of 80 g·plant^−1^ of a 10N–2.2P–24.9K plus micronutrients fertiliser (Hakaphos Naranja; Compo Agricultura, Barcelona, Spain) distributed through the entire period of cultivation. Plants were trained with bamboo canes. Weeds were removed manually, and no phytosanitary treatments were performed throughout the cultivation period (May-October 2016), as pest levels were below treatment limits.

### Characterization of plants and fruits

Plants were characterized using 14 morphological and agronomic descriptors based on EGGNET [42,43] and [41] descriptors: Plant Height (cm), Stem Diameter (mm), Leaf Pedicel Length (cm), Leaf Blade Length (cm), Leaf Blade Width (cm), Number of Flowers per Inflorescence, Corolla Diameter (mm), Fruit Pedicel Length (mm), Fruit Pedicel Diameter (mm), Fruit Length (cm), Fruit Width (cm), Fruit Calyx Prickles (measured in a scale from 0 = none to 9 = more than 30 prickles), Fruit Weight (g), and Yield (measured as the total weight of commercial fruits; kg/plant). Except for Plant Height and Stem Diameter, where only one data could be obtained per plant, for the remaining characters, at least five measurements were taken per plant. For fruit morphometric analysis, five fruits per replication were collected at a commercially ripe stage (i.e., physiologically immature) and were cut longitudinally and scanned using an HP Scanjet G4010 Photo Scanner (Hewlett-Packard, Palo Alto, CA, USA) at a resolution of 300 dpi. Scanned images were processed for fruit morphometric analysis with the fruit shape phenomics tool Tomato Analyzer version 4 software [44]. A total of 14 fruit morphometric descriptors were recorded using this tool: Perimeter (cm), Area (cm^2^), Width Mid-height (cm), Maximum Width (cm), Height Mid-width (cm), Maximum Height (cm), Curved Height (cm), Fruit Shape Index External I, Fruit Shape Index External II, Curved Fruit Shape Index, Proximal Fruit Blockiness, Distal Fruit Blockiness, Fruit Shape Triangle, and Fruit Shape Index Internal. A full description of the Tomato Analyzer traits measured can be found in Kaushik et al. [43] and Hurtado el al. [45].

### Morphological and agronomic data analysis

For each trait measured, the mean and range were calculated for the parental (n = 10) and hybrid (n = 45) groups. Mean values of parents and hybrids were compared with *t*-tests to detect differences among the two groups. The significance of differences among group means was evaluated using at *p <* 0.05 using the Statgraphics Centurion XVI software (StatPoint Technologies, Warrenton, VA, USA). The general combining ability (GCA) of parents and specific combining ability (SCA) of individual hybrids, along with the variance components, and narrow (*h*^*2*^) and broad (*H*^*2*^) sense heritabilities were estimated based on the Griffing’s [25] Method 2 Model 1 (fixed effects) using AGD-R (Analysis of Genetic Designs with R) software package [52]. The relative importance of GCA over SCA (GCA/SCA ratio) was estimated as GCA/SCA = 2 × s^2^_GCA_ / ((2 × s^2^_GCA_) + s^2^_SGA_) [26], Relative SCA values of individual hybrids were expressed in percentage (%) over the average of the trait. Pair-wise Pearson linear coefficients of correlation (*r*) were calculated using the Statgraphics Centurion XVI software, and significance of correlations was evaluated using the Bonferroni test [53]. The F1 hybrids heterosis over mid parent (*Het*; %) was calculated using formula *Het* = 100 × ((F1 − MP)/MP), where F1 = hybrid mean, and MP = mean of the parents.

### Genetic distances and correlation with hybrid performance and genetic parameters

Polymorphism information for the parental accessions used in this study was retrieved from genotyping data obtained by Acquadro et al. [17], where the 10 accessions used here were genotyped using a modified RAD sequencing approach targeting coding sequences. The VCF (Variant Call Format) file of Acquadro et al. [17], which consisted of 75,399 polymorphic sites, was filtered selecting only our 10 accessions. Subsequently, all missing data were excluded when individual accessions were compared to the reference genome of accession '67/3' developed by the Italian Eggplant Genome Sequencing Consortium [54]. Finally, all the non-polymorphic SNPs among the accessions were removed, yielding a total of 7,335 polymorphic SNPs in our set of accessions. The genetic distance (GD) among parents was calculated based on identity-by-state (IBS) as GD = 1-IBS using the TASSEL software version 5.0 Standalone [55]. The VCF file was exported to R software (version 1.1.383) using the vcfR package [56] and transformed into a genlight object using a vcfR2genlight function. A dendrogram with 1,000 bootstrap replicates was calculated using the aboot function of the popper package (version 2.6.1, https://cran.r-project.org/web/packages/poppr/index.html) using a UPGMA hierarchical clustering method and Hamming distance (bitwise distance). The relationship among GD of parents of individual hybrids was used to estimate pairwise Pearson linear correlations between GD and hybrid trait values, heterosis, and SCA.

## Results

### Variation in parents and hybrids

A wide variation was found for most of the traits evaluated both in the parents and their respective hybrids (Tables 2 and S1). In this way, differences over four-fold both in parents and hybrids groups were found for four conventional descriptors (Number of Flowers per Inflorescence, Fruit length, Fruit weight, and Yield), and for five Tomato Analyzer fruit descriptors (Area, Fruit Shape Index External I, Fruit Shape Index External II, Curved Fruit Shape Index, and for Fruit Shape Index Internal). For all traits an overlap in the ranges of variation was found between parent and hybrid groups (Table 2). Significant differences among averages of parents and hybrids (*p* < 0.05) were found only for Plant Height, Stem Diameter, and Yield, with higher values in hybrids (Table 2).

**Table 2:** Average values and ranges of variation. Traits evaluated include conventional morphological and Tomato Analyzer descriptors in eggplant parents and their hybrids. Probability of the *t*-test for comparison between parent and hybrid means is also included.

Descriptors	Parents (n = 10)		Hybrids (n = 45)		Prob. *t*
	Mean	Range	Mean	Range	
*Conventional descriptors*					
Plant Height (cm)	72.71	(65.80–85.63)	86.26	(59.80–121.50)	0.0069
Stem Diameter (mm)	14.95	(11.43–17.33)	17.90	(12.83–23.67)	0.0021
Leaf Pedicel Length (cm)	8.13	(4.83–12.04)	8.31	(5.45–12.24)	0.7573
Leaf Blade Length (cm)	23.91	(14.96–28.47)	25.34	(18.85–30.80)	0.1902
Leaf Blade Width (cm)	16.4	(10.80–22.83)	17.44	(14.22–23.22)	0.1940
Number of Flowers per Inflorescence	3.28	(1.00–5.33)	4.24	(1.00–7.00)	0.0551
Corolla Diameter (mm)	31.80	(19.37–43.00)	35.93	(23.50–47.17)	0.0603
Fruit Pedicel Length (mm)	50.92	(23.40–93.43)	42.80	(21.67–70.10)	0.0816
Fruit Pedicel Diameter (mm)	13.70	(7.50–21.33)	13.87	(7.83–20.37)	0.8819
Fruit Length (cm)	9.64	(4.40–19.20)	9.26	(3.73–18.87)	0.7647
Fruit Width (cm)	6.61	(3.83–9.83)	6.74	(4.13–10.10)	0.8081
Fruit Weight (g)	160.1	(55.2–245.7)	205.8	(63.6–353.3)	0.1123
Fruit Calyx Prickles^a^	1.00	(0.00–5.00)	1.25	(0.00–5.00)	0.6050
Yield (kg/plant)	2.38	(0.93–4.55)	3.28	(1.69–6.91)	0.0181
*Tomato Analyzer descriptors*					
Perimeter (cm)	26.76	(16.43–37.63)	30.88	(19.51–47.26)	0.0665
Area (cm^2^)	43.26	(17.48–78.88)	58.11	(24.13–100.91)	0.0464
Width Mid-height (cm)	6.20	(2.27–10.56)	6.83	(4.03–11.60)	0.3633
Maximum Width (cm)	6.50	(3.53–10.60)	7.00	(4.22–11.61)	0.4475
Height Mid-width (cm)	8.47	(4.32–13.66)	10.33	(4.95–19.66)	0.0847
Maximum Height (cm)	8.76	(4.68–13.90)	10.54	(5.04–19.93)	0.1006
Curved Height (cm)	9.15	(4.92–14.76)	11.06	(5.28–20.28)	0.0792
Fruit Shape Index External I	1.51	(0.69–3.33)	1.62	(0.78–3.40)	0.6683
Fruit Shape Index External II	1.70	(0.99–5.01)	1.65	(0.75–3.74)	0.9040
Curved Fruit Shape Index	1.84	(0.75–5.70)	1.76	(0.90–3.80)	0.7901
Proximal Fruit Blockiness	0.60	(0.46–0.76)	0.60	(0.30–0.75)	0.9462
Distal Fruit Blockiness	0.74	(0.64–0.97)	0.72	(0.61–0.90)	0.4744
Fruit Shape Triangle	0.83	(0.60–1.19)	0.85	(0.42–1.13)	0.7568
Fruit Shape Index Internal	1.69	(0.66–5.05)	1.66	(0.75–3.74)	0.9022

^a^Measured in a scale (0 = none; 9 = more than 30).

### GCA and SCA

The analysis of variance performed on the 55 genotypes (10 parents and 45 hybrids) detected no significant (*p* < 0.05) block effects except for Leaf Pedicel Length, Leaf Blade Length, Leaf Blade Width, Corolla Diameter, and Yield (Table 3). However, highly significant differences (*p* < 0.001) were found among genotypes for all traits. Similarly, highly significant (*p* < 0.001) effects were found for general combining ability (GCA) and specific combining ability (SCA) for all the traits evaluated, although higher values for the mean squares were observed for GCA than for SCA (Table 3). The GCA/SCA ratio was very variable, ranging from 0.15 for Yield to 4.08 for Fruit Shape Index External I. High (above 2) GCA/SCA ratios were found for conventional descriptors Leaf Blade Width, Fruit Pedicel Diameter, Fruit Length, and Tomato Analyzer descriptors Width Mid-height, Maximum Width, Fruit Shape Index External I, Fruit Shape Index External II, Curved Fruit Shape Index, and Fruit Shape Index Internal, while low (below 0.5) GCA/SCA ratios were found for conventional descriptors Stem Diameter, Fruit Calyx Prickles and Yield and for Tomato Analyzer descriptors Proximal Fruit Blockiness and Fruit Shape Triangle (Table 3). Narrow sense heritability (*h*^*2*^) values ranged between 0.11 for Proximal Fruit Blockiness and 0.83 for three fruit shape descriptors (Fruit Shape Index External I, Fruit Shape Index External II and Fruit Shape Index Internal). Traits related to plant vigour (like Plant Height, and Stem Diameter), Fruit Calyx Prickles, and Yield had low *h*^*2*^ values, while most fruit size and shape traits, either using conventional or Tomato Analyzer descriptors, had *h*^*2*^ values above 0.5 (Table 3). Broad sense heritability (*H*^*2*^) had values above 0.5 for all traits, except for Proximal Fruit Blockiness (0.42) and Fruit Shape Triangle (0.35). Most traits related to vigour, yield and fruit size and shape had values above 0.85 for *H*^*2*^ (Table 3). For traits with higher GCA/SCA ratios, the *h*^*2*^ and *H*^*2*^ values were much more similar than those with low GCA/SCA ratios (Table 3).

**Table 3:** Mean squares, GCA/SCA ratio [26], and narrow sense (*h*^*2*^) and broad sense heritabilities (*H*^*2*^) for the ANOVA for conventional morphological and Tomato Analyzer fruitdescriptors. Materials evaluated include 10 parents and 45 hybrids of eggplant. Griffing’s (1956) Method 2 Model 1 (fixed effects) of diallel analysis was used.

Mean squares								
Descriptors	Block^a^	Genotypes^a^	GCA^a^	SCA^a^	Error	GCA/SCA	*h*^*2*^	*H*^*2*^
d.f.	2	54	9	45	108			
*Conventional descriptors*								
Plant Height (cm)	105.07	643.47^***^	2035.18^***^	365.13^***^	71.41	0.56	0.39	0.74
Stem Diameter (mm)	4.72	23.65^***^	55.94^***^	17.20^***^	1.84	0.29	0.30	0.82
Leaf Pedicel Length (cm)	7.80^*^	8.39^***^	32.85^***^	3.50^**^	1.95	1.66	0.41	0.53
Leaf Blade Length (cm)	24.70^***^	28.85^***^	129.03^***^	8.82^***^	3.25	1.88	0.58	0.73
Leaf Blade Width (cm)	14.40^*^	15.61^***^	68.37^***^	5.05	3.71	4.01	0.46	0.52
Number of Flowers per Inflorescence	0.042	6.23^***^	20.70^***^	3.33^***^	0.02	0.52	0.51	0.99
Corolla Diameter (mm)	28.11^**^	120.00^***^	403.55^***^	63.29^***^	6.24	0.58	0.47	0.87
Fruit Pedicel Length (mm)	11.26	534.34^***^	2027.87^***^	235.63^***^	8.36	0.74	0.57	0.96
Fruit Pedicel Diameter (mm)	1.97^**^	33.30^***^	171.44^***^	5.67^***^	1.17	3.15	0.78	0.90
Fruit Length (cm)	1.29	38.47^***^	204.26^***^	5.31^***^	0.72	3.69	0.83	0.95
Fruit Width (cm)	0.18	7.24^***^	34.16^***^	1.86^***^	0.39	1.92	0.68	0.86
Fruit Weight (g)	73.45	20241.37^***^	86296.35^***^	7030.37^***^	457.60	1.09	0.64	0.94
Fruit Calyx Prickles	0.01	5.67^***^	16.63^***^	3.48^***^	0.01	0.40	0.44	1.00
Yield (kg/plant)	1.05^**^	3.68^***^	5.73^***^	3.26^***^	0.17	0.15	0.21	0.89
*Tomato Analyzer descriptors*								
Perimeter (cm)	1.04	124.13^***^	476.98^***^	53.56^***^	8.67	0.87	0.52	0.83
Area (cm)	9.03	1377.23^***^	5035.12^***^	645.65^***^	125.62	0.79	0.48	0.78
Width Mid-height (cm)	0.03	11.67^***^	59.14^***^	2.18^***^	0.57	3.04	0.75	0.87
Maximum Width (cm)	0.02	10.65^***^	53.21^***^	2.13^***^	0.58	2.82	0.73	0.86
Height Mid-width (cm)	0.39	28.51^***^	123.36^***^	9.54^***^	1.15	1.21	0.63	0.89
Maximum Height (cm)	0.37	28.79^***^	125.40^***^	9.47^***^	1.23	1.26	0.63	0.89
Curved Height (cm)	0.34	28.86^***^	120.65^***^	10.50^***^	1.22	1.07	0.61	0.89
Fruit Shape Index External I	0.01	1.44^***^	7.67^***^	0.19^***^	0.03	4.08	0.83	0.93
Fruit Shape Index External II	0.00	2.21^***^	11.79^***^	0.30^***^	0.05	3.93	0.83	0.94
Curved Fruit Shape Index	0.01	2.42^***^	12.52^***^	0.40^***^	0.06	3.07	0.80	0.93
Proximal Fruit Blockiness	0.01	0.03^***^	0.04^***^	0.02^***^	0.01	0.18	0.11	0.42
Distal Fruit Blockiness	0.00	0.02^***^	0.07^***^	0.01^***^	0.00	1.23	0.45	0.63
Fruit Shape Triangle	0.00	0.07^***^	0.12^***^	0.06^***^	0.03	0.26	0.12	0.35
Fruit Shape Index Internal	0.00	2.25^***^	11.96^***^	0.30^***^	0.05	3.87	0.83	0.94

^a***^, ^**^, ^*^ indicate significant at *p* < 0.001, *p* < 0.01, or *p* < 0.05, respectively.

For all traits, a significant GCA effect was detected in most of the parents, except for Leaf Blade Width and Proximal Fruit Blockiness where five and eight parents, respectively, did not present GCA effects (Table 4). Regarding outstanding GCA values, the flattened accession A0416 was characterized by strikingly low GCA values for Plant height, Stem Diameter, and for high absolute GCA values for fruit traits associated to flattened fruits (Table 4); the Spanish landrace AN-S-26 for high GCA values of Fruit Pedicel Diameter; the round accession ASI-S-1 for high GCA values for Fruit Area and for traits associated to fruit width, as well as for high GCA absolute values for fruit shape traits associated to elongated shape, and low GCA values for Yield and for Number of Flowers per Inflorescence; the élite background accession DH621 for high GCA values for Fruit Weight and fruit Area; the pickling accession H15 for high GCA for Fruit Pedicel Length; the striped accession IVIA371 for high GCA values for Fruit Calyx Prickles and Yield, and low GCA values for Plant Height; the white accession MEL1 did not present particularly high or low levels for any trait; the mauve-colored accession MEL5 had high GCA levels for the Number of Flowers per Inflorescence, and low GCA values for Fruit Weight and fruit Area; the elongated accession MM1597 for its high GCA values for Plant Height, leaf size traits, fruit length, and for fruit shape traits associated to elongated fruits, and Yield, while it had low GCA values for traits associated to fruit width; finally, the weedy accession INS2 in general displayed low GCA levels for Corolla Diameter, traits related to leaf and fruit size, and Yield (Table 4).

**Table 4:** General combining ability estimates of parents for conventional morphological and Tomato Analyzer fruit descriptors for the 10 eggplant parents evaluated.

Descriptors^a^	*S*. *melongena*									*S*. *insanum*
	A0416	ANS26	ASIS1	DH621	HI5	IVIA371	MEL 1	MEL5	MM1597	INS2
*Conventional descriptors*										
Plant Height (cm)	-13.26^***^	5.56^***^	3.13^*^	5.87^***^	4.11^**^	-9.75^***^	-4.96^***^	-1.71	10.87^***^	0.15
Stem Diameter (mm)	-1.79^***^	0.08	-1.18^***^	-0.27	-0.47	-0.18	0.55^*^	-0.65^**^	1.35^***^	2.55^***^
Leaf Pedicel Length (cm)	-1.07^**^	0.96^***^	-0.23	0.74^**^	0.66^*^	1.07^***^	-0.7^**^	0.22	0.18	-1.84^***^
Leaf Blade Length (cm)	-1.65^***^	0.55	1.50^***^	2.44^***^	-0.09	0.28	0.03	-1.00^**^	1.97^***^	-4.01^***^
Leaf Blade Width (cm)	-1.38^***^	-0.07	0.55	0.38	0.05	0.37	0.27	-0.96^**^	2.98^***^	-2.18^***^
Number of Flowers per Inflorescence	-0.12^***^	-0.96^***^	-1.31^***^	-0.23^***^	-0.25^***^	0.27^***^	0.24^***^	0.86^***^	1.21^***^	0.29^***^
Corolla Diameter (mm)	-0.52	2.32^***^	-1.34^**^	-1.42^**^	3.18^***^	4.45^***^	1.98^***^	-2.90^***^	1.18^*^	-6.92^***^
Fruit Pedicel Length (mm)	-10.65^**^	5.97^***^	-6.20^***^	6.20^***^	12.55^***^	0.97	0.87	-1.47^***^	2.42^***^	-10.67^***^
Fruit Pedicel Diameter (mm)	-2.52^***^	3.11^***^	0.64^***^	1.32^***^	2.72^***^	1.32^***^	-0.43^*^	-2.44^***^	-0.74^***^	-2.98^***^
Fruit Length (cm)	-2.88^***^	0.03	-1.81^***^	1.75^***^	0.15	1.02^***^	0.19	0.17	4.83^***^	-3.46^***^
Fruit Width (cm)	0.99^***^	0.81^***^	1.00^***^	0.48^***^	-0.02	0.56^***^	0.16	-1.20^***^	-1.22^***^	-1.55^***^
Fruit weight (g)	1.09	17.45^***^	17.37^***^	58.00^***^	31.66^***^	35.06^***^	8.92^*^	-55.45^***^	-3.62	-110.47^***^
Fruit Calyx Prickles	-0.02	-0.27^***^	-0.36^***^	0.06^***^	0.17^***^	1.81^***^	-0.02	-0.36^***^	-0.52^***^	-0.49^***^
Yield (kg/plant)	-0.31^***^	-0.22^**^	-0.54^***^	0.35^***^	-0.20^**^	0.43^***^	0.31^***^	0.18^*^	0.51^***^	-0.51^***^
*Tomato Analyzer descriptors*										
Perimeter (cm)	-2.76^***^	1.55^**^	1.67^**^	3.38^***^	1.62^**^	2.15^***^	-0.33	-3.76^***^	4.04^***^	-7.56^***^
Area (cm^2^)	-7.56^***^	9.01^***^	10.66^***^	10.34^***^	6.88^***^	8.91^***^	-1.18	-15.26^***^	1.37	-23.17^***^
Width Mid-height (cm)	1.04^***^	1.00^***^	2.18^***^	0.01	0.29^*^	0.58^***^	-0.44^***^	-1.63^***^	-1.72^***^	-1.31^***^
Maximum Width (cm)	0.91^***^	0.93^***^	2.06^***^	0.13	0.31^*^	0.55^***^	-0.39^***^	-1.70^***^	-1.41^***^	-1.40^***^
Height Mid-width (cm)	-2.69^***^	0.21	-1.01^***^	1.88^***^	0.75^***^	0.80^***^	0.34	-0.57^**^	3.09^***^	-2.79^***^
Maximum Height (cm)	-2.61^***^	0.16	-0.97^***^	1.84^***^	0.72^***^	0.74^***^	0.33	-0.59^**^	3.23^***^	-2.86^***^
Curved Height (cm)	-2.36^***^	0.25	-0.69^***^	1.94^***^	0.72^***^	0.76^***^	0.16	-0.78^***^	3.05^***^	-3.05^***^
Fruit Shape Index External I	-0.60^***^	-0.22^***^	-0.52^***^	0.22^***^	0.00	-0.06	0.08^*^	0.36^***^	0.99^***^	-0.25^***^
Fruit Shape Index External II	-0.69^***^	-0.27^***^	-0.59^***^	0.23^***^	-0.02	-0.09^*^	0.06	0.34^***^	1.33^***^	-0.30^***^
Curved Fruit Shape Index	-0.65^***^	-0.28^***^	-0.57^***^	0.24^***^	-0.04	-0.11^*^	0.02	0.31^***^	1.42^***^	-0.33^***^
Proximal Fruit Blockiness	0.03	-0.05^**^	0.00	-0.03	0.01	0.00	-0.02	0.02	0.07^***^	-0.02
Distal Fruit Blockiness	-0.06^***^	-0.04^***^	-0.05^***^	0.03^***^	0.01	-0.01	0.03^***^	0.01	0.08^***^	-0.02
Fruit Shape Triangle	0.12^***^	-0.03	0.06^*^	-0.07^*^	-0.01	0.01	-0.07^*^	0.00	0.00	-0.02
Fruit Shape Index Internal	-0.69^***^	-0.27^***^	-0.60^***^	0.23^***^	-0.02	-0.09^*^	0.06	0.34^***^	1.34^***^	-0.31^***^

^a***^, ^**^, ^*^ indicate significant at *p* < 0.001, *p* < 0.01, or *p* < 0.05 respectively.

The range of variation for SCA values with respect to means for individual descriptors is given in Table 5. The lowest SCA range among hybrids for SCA was for Leaf Blade Width (-9.98% to 14.00%), while the highest was found for Fruit Calyx Prickles (-139.00% to 198.11%). However, for most traits the SCA values ranged between -50% and 50% (Table 5). Other traits, apart from Fruit Calyx Prickles, with values outside these ranges were Yield, with values between -41.77% and 87.20%, and several Tomato Analyzer descriptors like Area, Height Mid-width, and Maximum Height with positive values above 50%, and Fruit Shape Index External II, curved Fruit Shape Index, Proximal Fruit Blockiness, and Fruit Shape Triangle with negative values below -50% (Table 5). In general, traits with high absolute value for the SCA range had low GCA/SCA ratios (Table 3).

**Table 5:** Range of specific combining ability (SCA) estimates. Values are expressed as percentage over the mean of the 45 hybrids obtained among 10 eggplant parents.

Traits	SCA values (% over mean)	
	Minimum	Maximum
*Conventional descriptors*		
Plant Height (cm)	-19.14	26.35
Stem Diameter (mm)	-22.74	23.13
Leaf Pedicel Length (cm)	-20.94	33.93
Leaf Blade Length (cm)	-10.50	20.01
Leaf Blade Width (cm)	-9.98	14.00
Number of Flowers per Inflorescence	-38.45	47.65
Corolla Diameter (mm)	-23.32	21.23
Fruit Pedicel Length (mm)	-47.78	41.31
Fruit Pedicel Diameter (mm)	-21.26	17.80
Fruit Length (cm)	-23.76	49.14
Fruit Width (cm)	-19.14	27.15
Fruit Weight (g)	-28.13	41.78
Fruit Calyx Prickles	-139.00	198.11
Yield (kg/plant)	-41.77	87.20
*Tomato Analyzer descriptors*		
Perimeter (cm)	-19.01	37.15
Area (cm^2^)	-32.87	58.54
Width Mid-height (cm)	-19.61	25.03
Maximum Width (cm)	-18.56	24.42
Height Mid-width (cm)	-20.43	56.46
Maximum Height (cm)	-20.70	54.66
Curved Height (cm)	-21.98	52.46
Fruit Shape Index External I	-30.90	43.27
Fruit Shape Index External II	-49.00	36.30
Curved Fruit Shape Index	-58.61	31.86
Proximal Fruit Blockiness	-54.93	19.97
Distal Fruit Blockiness	-16.68	15.30
Fruit Shape Triangle	-70.90	24.82
Fruit Shape Index Internal	-49.50	27.76

### Correlations among traits

One hundred twenty-two out of the 378 pair-wise correlations (32.3%) among traits were significant according to the Bonferroni test (*p* < 0.05; *r* ≥ 0.4928) (S2 Table). Only 16 out of the 122 significant correlations (13.1%) had negative values. For 24 of the positive correlations, *r* values were higher than 0.8. In general, descriptors related to leaf size, Corolla Diameter, fruit size, elongated shapes and Yield were found to be significantly correlated, although Yield was not correlated with Fruit Weight (S2 Table). Many significant correlations were found among traits related to fruit shape, both for conventional and Tomato Analyzer descriptors. Also, a negative correlation was found between the Number of Flowers per Inflorescence and fruit width traits (S2 Table).

### Genetic distances and correlation with hybrid performance and genetic parameters

**Fig 2:**
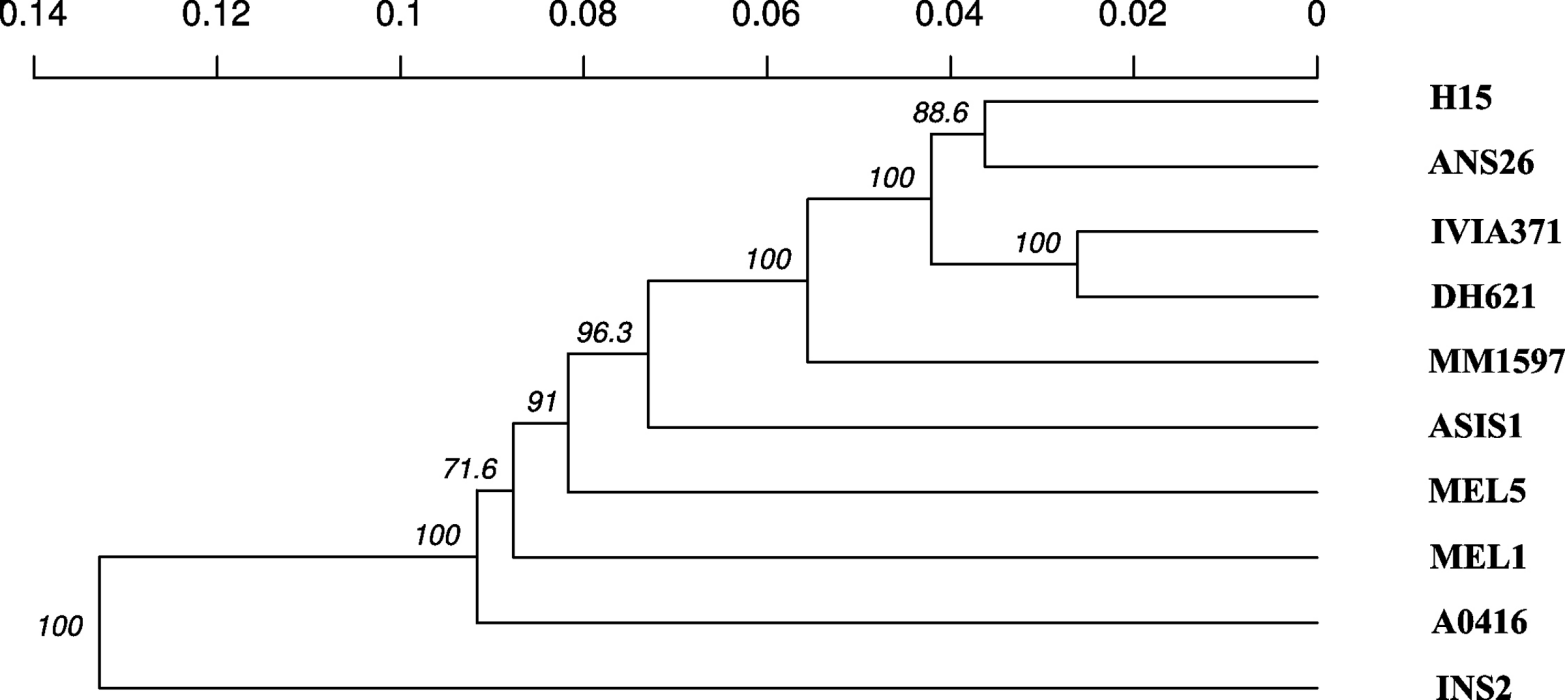
UPGMA dendrogram displaying relationships of nine *S*. *melongena* (A0416, ANS26, ASIS1, DH621, H15, IVIA371, MEL1, MEL5, and MM1597) and one *S*. *insanum* (INS2) accessions based on 7,335 polymorphic SNPs. Phenetic relationships among accessions were derived from Hamming distance (bitwise distance). Bootstrap values (based on 1000 replications; expressed in percentage) are indicated at the corresponding nodes.

The genetic distance (GD) based on 7,335 coding SNPs ranged from GD = 0.0094 between DH621 and IVIA-371 to a maximum value of GD = 0.0389 between MEL1 and INS2 (S3 Table). The highest values for GD (above 0.03) were found between the weedy *S*. *insanum* INS2 and all the cultivated accessions, and also between the cultivated A0416 and MEL1 accessions (S3 Table). The lowest values of GD were found among the three Spanish landraces (AN-S-26, H15, IVIA-371) and among the latter and the élite line DH621, which in all cases had GD values below 0.015 (S3 Table). These results are confirmed in the cluster analysis dendrogram, which shows that *S*. *insanum* INS2 is basal to the *S*. *melongena* accessions, while the three Spanish and the élite line DH621 cluster together (Fig 2).

When considering the 45 hybrids among all parents, including the weedy INS2, GD was significantly correlated (*p* < 0.05) with hybrid’s trait values for 14 traits out of a total of 28 (Table 6). However, significant *r* values between GD and hybrid’s trait values were generally low, with absolute values always below 0.5. Most of the significant correlations with GD were negative, including traits like leaf size descriptors, fruit size descriptors and Yield. The correlations between GD and trait heterosis (*Het*) generally were non-significant, and only significant positive correlations with GD were found for Leaf Blade Width and Fruit Pedicel Diameter, while negative correlations were found for Proximal Fruit Blockiness and Fruit Shape Triangle. Regarding the correlations between GD and SCA the only significant correlation (negative) was observed for Fruit Weight (Table 6). Given that the inclusion in these correlation analyses of the weedy INS2, which presents high GD values with the other accessions, might distort the results, we performed the same analysis using only the nine cultivated accessions and their respective 36 hybrids. The results obtained were similar to those obtained with all the accessions with some variations (Table 6). In this way, a negative correlation between GD and hybrid value for Plant Height, which was non-significant when considering all accessions, was found to be significant when excluding INS2. However, the negative correlations between GD and hybrid values for Corolla Diameter, Fruit Pedicel Length, Yield, and Curved Fruit Shape Index, which were significant in the analysis with all accessions, were not significant when only *S*. *melongena* accessions are considered. Regarding the relationship between GD and *Het* in the *S*. *melongena* accessions, the same significant correlations were detected than when all the materials are included in the analyses, except for a new significant positive correlation between GD and Stem Diameter. Finally, no significant correlations were detected between GD and SCA for the *S*. *melongena* materials (Table 6).

**Table 6:** Correlations between genetic distances among parents and hybrid trait values, heterosis (*Het*), and specific combining ability (SCA). Results are presented considering the 10 eggplant parents (nine cultivated *S*. *melongena* and one weedy *S*. *insanum*; n = 45 hybrids), and only the nine *S*. *melongena* parents (n = 36 hybrids).

Traits	All parents			Only *S*. *melongena* parents		
	Trait^a^	*Het*^a^	SCA^a^	Trait^a^	*Het*^a^	SCA^a^
*Conventional descriptors*						
Plant Height (cm)	-0.292	-0.016	0.126	-0.388^*^	0.005	0.127
Stem Diameter (mm)	0.021	0.143	0.309^*^	0.066	0.452^**^	0.278
Leaf Pedicel Length (cm)	-0.468^***^	0.131	-0.182	-0.408^**^	0.074	-0.210
Leaf Blade Length (cm)	-0.558^***^	0.200	-0.008	-0.491^**^	-0.124	-0.056
Leaf Blade Width (cm)	-0.377^**^	0.359	-0.085	-0.193	0.172	-0.219
Number of Flowers per Inflorescence	0.099	-0.084	0.021	0.164	-0.094	-0.037
Corolla Diameter (mm)	-0.318^*^	0.200	-0.072	-0.242	0.007	-0.099
Fruit Pedicel Length (mm)	-0.356^*^	0.257	-0.094	-0.304	0.196	-0.198
Fruit Pedicel Diameter (mm)	-0.397^**^	0.338^*^	-0.074	-0.352^*^	0.331^*^	-0.109
Fruit Length (cm)	-0.470^***^	0.060	-0.188	-0.341^*^	0.040	-0.113
Fruit Width (cm)	-0.231	0.094	-0.137	-0.111	0.044	-0.008
Fruit Weight (g)	-0.450^***^	0.034	-0.438^**^	-0.391^*^	-0.035	-0.321
Fruit Calyx Prickles	-0.057	0.016	-0.157	-0.092	0.035	-0.223
Yield (kg/plant)	-0.296^*^	0.190	0.152	-0.137	0.116	0.140
*Tomato Analyzer descriptors*						
Perimeter (cm)	-0.471^***^	0.078	-0.183	-0.372^*^	0.019	-0.121
Area (cm^2^)	-0.404^**^	0.098	-0.315^*^	-0.383^*^	0.017	-0.276
Width Mid-height (cm)	-0.109	0.121	-0.248	-0.143	0.009	-0.246
Maximum Width (cm)	-0.131	0.166	-0.230	-0.160	0.038	-0.235
Height Mid-width (cm)	-0.453^***^	0.070	-0.186	-0.338^*^	0.049	-0.137
Maximum Height (cm)	-0.448^***^	0.074	-0.174	-0.335^*^	0.028	-0.128
Curved Height (cm)	-0.456^***^	0.093	-0.196	-0.354^*^	0.030	-0.153
Fruit Shape Index External I	-0.283	-0.063	0.058	-0.158	-0.009	0.104
Fruit Shape Index External II	-0.280	-0.052	0.086	-0.153	0.050	0.100
Curved Fruit Shape Index	-0.286^*^	-0.022	0.068	-0.164	0.031	0.064
Proximal Fruit Blockiness	0.010	-0.286^*^	-0.247	0.067	-0.375^*^	-0.173
Distal Fruit Blockiness	-0.106	0.171	0.041	0.048	0.193	0.012
Fruit Shape Triangle	0.060	-0.347^*^	-0.228	0.028	-0.444^**^	-0.151
Fruit Shape Index Internal	-0.281	-0.033	0.082	-0.154	0.049	0.100

^a***^, ^**^, ^*^ indicate significant at *p* < 0.001, *p* < 0.01, or *p* < 0.05, respectively.

## Discussion

F1 hybrids are often heterotic and generally present a better performance than non-hybrid varieties under sub-optimal conditions [11,57]. Therefore, F1 hybrid breeding is one of the most employed strategies for vegetable crops breeding. Selection of parents giving hybrids with improved performance is one of the challenges faced by breeders [23,24]. In this way, knowledge of values of genetic parameters for traits with agronomic relevance, including the contribution of additive and non-additive effects, heritability values, and correlations among them provides important information for identifying appropriate parental combinations [38]. In addition, given that, the number of potential hybrid combinations grows exponentially with increasing number of parents, tools that allow predicting hybrid performance facilitate the selection of parents [27].

In several crops, genetic distances among parents have been proved useful to predict the performance of hybrids, although the results depend on the crop, the diversity present in the parents, and the markers used [28,39,40,58,59]. In eggplant, to our knowledge, a single work studied the relationship between genetic distances, based on AFLP markers, of parents and yield and fruit weight of hybrids using Spanish local varieties as parents [39]. These authors found relatively high correlations (*r* > 0.6) between parents genetic distance and yield or fruit weight of hybrids, although their results were based on just 10 hybrids. Our work tried to provide an integrated perspective for obtaining relevant information for the eggplant breeding. We used a half-diallel analysis, which gives information on the magnitude of general and specific combining abilities and trait heritabilities [25–27], using 10 parents from different genetic backgrounds, origins, and morphological characteristics. The parents and hybrids were characterized for a wide number of traits of agronomic interest and fruit shape, and the potential of genetic distances among parents for predicting hybrid performance, heterosis and SCA was evaluated. To our knowledge this is the most comprehensive work done so far for devising strategies for the selection of parents for hybrids development in eggplant.

We found that parents and hybrids displayed wide and overlapping ranges of variation, as compared with other works evaluating the diversity for morphological and agronomic traits of eggplant [43,45,60,61]. However, despite the wide diversity, on average the group of hybrids had significantly higher vigour (plant height and stem diameter) and yield than the group of parentals, supporting the claim that eggplant hybrids represent a productive advantage over non-hybrid varieties [5,11,62]. In fact, in our work, hybrids had an average yield over 1/3 higher than parents. This is in agreement with many other works that have found that eggplant hybrids frequently have a better agronomic performance than non-hybrid varieties [34–37,39,63], and therefore are of great interest for improving eggplant production.

The high diversity in the parental and hybrid materials for the traits evaluated was matched by significant GCA and SCA values for all traits, revealing the presence of significant additive and non-additive effects in all traits [26,27]. This suggests that a wide genetic variation exists for the traits evaluated among the parents included in the study. Wide variation in the GCA/SCA ratio among traits indicates that considerable differences exist among them in the gene action. In this way, traits with higher GCA/SCA ratios, like most of the fruit shape traits have a mostly additive genetic control as occurs in other crops such as tomato [64] or melon [65]. Traits with low GCA/SCA values have a predominantly non-additive (i.e., dominant, additive × dominant, and dominant × dominant effects) genetic control [26,27]. These traits with a higher relative proportion of SCA included several related to vigour (plant height and stem diameter), number of flowers per inflorescence, prickliness, and yield. Vigour traits in eggplant are heterotic both in intraspecific and interspecific crosses [36,37,39,43], indicating that this is a general phenomenon in the eggplant genepool. Regarding the number of flowers per inflorescence and prickliness both traits have been found to display significant heterosis in interspecific crosses [43]. While little information exists on the inheritance of the number of flowers per inflorescence in eggplant [66], prickliness has been described as a monogenic or oligogenic trait, with a mostly dominant genetic control, although in some interspecific crosses it is recessive [43,49,66]. Yield displayed the lowest levels for the GCA/SCA ratio, which is in agreement with other works in eggplant [67] and in other solanaceous fruit crops like tomato [68] or pepper [69], indicating that non-additive effects and their interactions play a major role in the genetic control of this trait. This further supports the development of hybrids as an appropriate strategy for enhancing eggplant yield [5,39]. For fruit size traits in general values revealed a similar effect of GCA and SCA, indicating that, as in other studies with eggplant [67], both additive and non-additive effects are important. This suggests that breeding for fruit size will require parents with good GCA values, but also specific hybrid combinations.

Broad-sense heritability (*H*^*2*^) values were generally high, indicating that most of the variation observed is genetically determined and that selection among varieties or hybrids will be efficient [38]. Probably the fact that the materials included encompassed a wide diversity for the traits evaluated also contributed to high *H*^*2*^ values. This is in agreement with Hurtado et al. [45], whom found high *H*^*2*^ values for eggplant fruit shape traits. However, when considering narrow-sense heritability (*h*^*2*^), which only takes into account additive variance, values were lower, especially for traits with lowest GCA/SCA ratios. In this way, *h*^*2*^ values for important agronomic traits, like those related to vigour, prickliness or yield were relatively low, difficulting genetic advances in breeding programmes [38]. However, Rodríguez-Burruezo et al. [39] found high correlation values between parental means and hybrid values (i.e., *h*^*2*^ values) in hybrids of Spanish local varieties. This may be an indication that heritability values in eggplant largely depend on the population evaluated. On the contrary, fruit shape traits generally had high values for *h*^*2*^, suggesting a high selection efficiency in breeding programmes.

High GCA values for traits of interest were scattered among different accessions, indicating that none of the accessions tested had the best combination of GCA values for traits of interest in breeding. For example, the Spanish local accession IVIA371 had high GCA values for Yield, which is a favourable trait, but also for the number of prickles, which is unfavourable [5]. Regarding other traits, like fruit shape, for which different shapes may be demanded by the markets [11], accessions A0416 and MM1597 had, respectively, low and high GCA values associated to elongated fruits, and may be parents of interest for specific markets demanding flattened or elongated fruits, respectively. As expected, the weedy *S*. *insanum* INS2 accession had low values of GCA for yield and fruit size traits. Although *S*. *insanum* is self-compatible with eggplant and hybrids are fully fertile [70], fruits are smaller, and yield is lower [43,48]. Amazingly, despite being a wild species from the “spiny” group of eggplant wild relatives [71], INS2 had a negative GCA value for the number of prickles. Although *S*. *insanum* is generally prickly, due to introgression and genetic flow between *S*. *melongena* and *S*. *insanum* [72], there is a continuum of *S*. *insanum* forms between highly prickly forms and non-prickly ones [48,73], and INS2 corresponds to the latter.

SCA values with respect to trait means were very variable, and generally higher in traits with low *h*^*2*^ values, like those related to plant vigour and yield. This suggests that for obtaining hybrids with high yield, many hybrids will have to be tested to identify good combinations. Also, high SCA values were observed for prickles. These results indicate that for these traits, obtaining good hybrids require specific combinations of parents, being the GCA values of the parents of lesser importance [25–27].

Positive correlations detected between traits related to leaf, flower and fruit size suggests that the size of these organs might have a common genetic or physiological basis, as has been found in tomato [74–76]. Yield was also positively correlated to leaf and flower size traits, but not to fruit size, indicating that high yields can be obtained even though fruits are not large, which would require higher fruit set ratios. Interestingly, the number of flowers per inflorescence was negatively correlated with wide fruits. In this respect, van der Knaap and Tanksley [77] found that in tomato the number of flowers per inflorescence and several fruit shape traits were correlated, which may suggest that a common hormonal control may affecting both traits. As expected and found in other eggplant works, most of the fruit shape traits were interrelated [45], suggesting that although a good characterization of eggplant fruit shape can be obtained with Tomato Analyzer software [43,45,78], good information on fruit shape in eggplant can be retrieved with a limited number of descriptors.

Genetic distances based on high-throughput SNP markers were largely in agreement with taxonomic relationships and origins [17,71]. In this way, *S*. *insanum*, which is the ancestor of *S*. *melongena* [48,73] was the genetically most distant accession compared to the others. This *S*. *insanum* accession was also basal to the *S*. *melongena* accessions in the cluster analysis dendrogram as found in the previous study of Acquadro et al. [17]. Interestingly, our study allowed clarifying relationships among *S*. *melongena* materials that were unresolved in the general study with a large number of accessions from *S*. *melongena* relatives [17]. The fact that the three Spanish accessions, together with the élite background breeding line DH621, derived from the commercial hybrid Ecavi, which is used in the Mediterranean region [11], cluster together is in agreement with a general genetic differentiation of the Mediterranean eggplants group [79, 80]. The fact that accession A0416 is basal to the other *S*. *melongena* accessions, and has a unique flattened shape, which is quite unusual in *S*. *melongena* [43,45], might be an indication of introgression with some related species, like *S*. *aethiopicum* group Kumba, which has flattened fruits [78], although further genotyping studies should be performed to confirm this hypothesis.

Correlations between genetic distances and hybrid trait values or parameters like heterosis or SCA can be very useful for selecting hybrids [81]. In our case, few correlations were observed between genetic distances among parents and hybrid trait values, heterosis, or SCA, independently if the weedy *S*. *insanum* was excluded from the correlation analyses or not. In any case, the significant correlation values obtained had a relatively low absolute value, with absolute values for *r* always below 0.5, and therefore having a limited predictive value. Negative correlations between hybrid values and genetic distance for leaf and fruit size traits is probably resulting from the fact that the two accessions with highest genetic distances with respect to the others (INS2 and A0416) are the ones with smallest leaves and fruits. Also, a negative correlation of genetic distance with yield when hybrids with all parental accessions are included is probably a consequence of the low yield of INS2. In fact, when hybrids with this parent are excluded this correlation is not significant. Traits for which genetic distances may have some predicting value are stem diameter, where a positive correlation is obtained with heterosis when all accessions are included, and with SCA when only *S*. *melongena* accessions are included. Overall, our results indicate that genetic distances based on coding SNP markers in the materials studied are of little predictive value. This is in contrast with a previous study of Rodríguez-Burruezo et al. [39], who found positive correlations between AFLP-based genetic distance and yield and fruit weight in 10 hybrids of Spanish local varieties. The fact that the materials used are very distinct, and much more diverse in our case, the markers used are different (AFLPs vs. SNPs) and the low number of hybrids evaluated in the study of Rodríguez-Burruezo et al. [39] might account for these differences. This suggests that differences in the predictive value of genetic distances for the performance, heteroris, or SCA of hybrids does not only depend on crops and markers used [28,31,32,40] but differences may also exist within a crop, as has been demonstrated for maize [58,81].

Overall our study provides relevant information for eggplant breeding, in particular for the development of improved F1 hybrids. The highly significant differences observed for GCA and SCA for all traits indicates that there is large genetic and gene action diversity in the set of parents and hybrids that can be exploited for breeding. The differences in GCA/SCA ratios and heritabilities, as well as correlations among traits also will condition the breeding strategy to be followed in order to maximize genetic gains in eggplant [38]. With our results we suggest that hybrids are a fast and appropriate strategy to develop improved eggplant cultivars. The fact that genetic distances among parents are not good predictors of the performance of eggplant hybrids indicates that many hybrid combinations may have to be tested to identify superior hybrids. It also suggests that other molecular techniques, like the use of markers linked to genes or QTLs controlling traits of interest [66], may be a more appropriate strategy for preselecting parents in eggplant hybrid breeding programs than the use of genetic distances among parents.

## Supporting information

S1 Table Values for each of the parents and hybrids for the 28 conventional and Tomato Analyzer descriptors evaluated. (XLSX)

S2 Table Pearson linear correlation coefficients between descriptors. Only those traits for which at least one correlation was significant (values in bold) according to the Bonferroni test (*p* < 0.05; *r* ≥ 0.4928) are included.
(XLSX)

S3 Table Genetic distance (GD) matrix among the 10 eggplant parents. GD values are based on identity by state (IBS) and calculated as GD = 1-IBS.
(XLSX)
